# Selective introduction of Cu impurity into fine-dispersed ZnS obtained during the process of one-stage synthesis

**DOI:** 10.1186/s11671-017-2274-7

**Published:** 2017-08-29

**Authors:** Y.Y. Bacherikov, O.B. Okhrimenko, A.G. Zhuk, R.V. Kurichka, A.V. Stronski, A.V. Gilchuk, M.V. Herkalyuk, V.V. Kidalov

**Affiliations:** 1 V. Lashkaryov Institute of Semiconductor Physics of the NASU, Pr. Nauky 41, Kyiv, 03028 Ukraine; 2 National Technical University of Ukraine “Igor Sikorsky Kyiv Polytechnic Institute”, Pr. Peremogy 37, Kyiv, 03506 Ukraine; 3 Berdyansk State Pedagogical University, 4, Shmidta str, Berdyansk, 71100 Ukraine

**Keywords:** ZnS, Self-propagating high-temperature synthesis, Retrograde solubility, Luminescence, X-ray spectroscopy

## Abstract

Fine ZnS:Cu, obtained by method of self-propagating high-temperature synthesis was investigated. As flux in the mixture NaCl was used, Zn and S were taken in stoichiometric ratio; Cu concentration in charge consisted ~1.5 wt.%. Using SEM data, it was established that obtained ZnS:Cu consists from two fractions—first with particles sizes ~10 μm and more, and other with sizes 50–500 nm. It was established that composition of ZnS:Cu fractions was essentially different. According to EDS data, Cu concentration in particles of fraction with 50–500 nm sizes consists ~2 wt.%, and in particles with sizes ~10 μm and more the presence of Cu was not detected. The reasons that lead to the selective doping of particles in dependence on their size and also the role of NaCl in processes undergoing during synthesis of material are discussed.

## Background

At the present time, considerable attention is devoted to the development of different new technological methods of complex semiconductor structure fabrication. In this connection, of particular interest are investigations concerning optimization of semiconductor material synthesis methods directed on fabrication of high quality (stoichiometric ones, without impurities, etc.) or complex semiconductor structures (solid solutions, nanostructured materials) in one technological cycle. Such investigations have also importance for fundamental knowledge because they enable to more deeply understand the interconnection between structure, composition, and properties of produced material and technological regimes of its synthesis. Regularity studies of mentioned above interconnection are important also as far as application possibilities are concerned, understanding of regularities in sequence «composition – structure – properties» enables to control or modify structure and properties of materials.

Particular place in investigations of fabrication regime influence on structure and properties of materials has the case when fusing agents are used in the synthesis of material. If it is necessary to obtain the substance in the form of well-formed crystals, the fluxes are often used as mineralizators. It is especially expedient in the high-temperature conditions when high mobility of atoms which form the lattice can lead to the formation of the big quantity of defects [1].

In this connection, is interesting to investigate peculiarities of possibility of ZnS fabrication obtained by method of self-propagating high-temperature synthesis (SHS) using as flux material with ion bonding. SHS technological possibilities are wide and enable to realize doping of material by different elements and compounds during synthesis process. Variation of methods of burning process in SHS wave provide possibility of fabrication of targeted product in the form of cast sample with preset sizes as well in the form of powder with necessary size dispersion.

It is necessary to note that the use of fluxes enables to control the temperature of burning process and to change dopant solubility conditions in the synthesized material. In particular, to provide conditions for so-called «retrograde solubility» [2], when introduced impurity either enters only in particular areas of material or do not enter at all. Such situation is realized when Fermi level intersects with the top of localized impurity states band.

Variation of methods of burning process in SHS wave provide possibility of fabrication of targeted product in the form of cast sample with preset sizes as well in the form of powder with necessary size dispersion.

This phenomenon will be better pronounced in materials with localized impurity state bands localized in maximal proximity to the Fermi level. Such materials balance on stability edge of covalent complexes of introduced and main metal [3].

This work was devoted to the clarification of NaCl influence introduced into mixture as a fusing agent, on the concentration of Cu in particles of fine-dispersed ZnS:Cu with different sizes, obtained by SHS method [4, 5].

## Methods

In present work investigations of fine ZnS:Cu obtained by SHS method (ZnS:Cu–SHS) were carried out. For fabrication of ZnS:Cu–SHS, Zn and S were taken in stoichiometric ratio. Doping by copper admixture was carried out from copper chloride CuCl directly during synthesis process. Concentration of Cu in charge consisted ~1.5 wt.%. ZnS:Cu was obtained at temperatures that provided process of interaction of sulfur and zinc. Part of the release during interaction reaction of S and Zn was absorbed by flux (NaCl), which enable to decrease the temperature of material synthesis. Quantity of NaCl in mixture consisted 5 wt.%.

Photoluminescence spectra (PL) and spectra of luminescent excitation (PLE) were registered at room temperature using SDL-2 installation. During PLE registration excitation was carried out using radiation of xenon lamp and MDR-12 monochromator.

Morphology and particle sizes studies were carried out using scanning electron microscope JAMP-9500F (Jeol).

Measurements of particles element composition were made by using energy dispersive X-ray spectroscopy (EDS) method and INCA PentaFETx3 (Oxford Instruments) spectrometer.

## Results and discussion

**Fig. 1:**
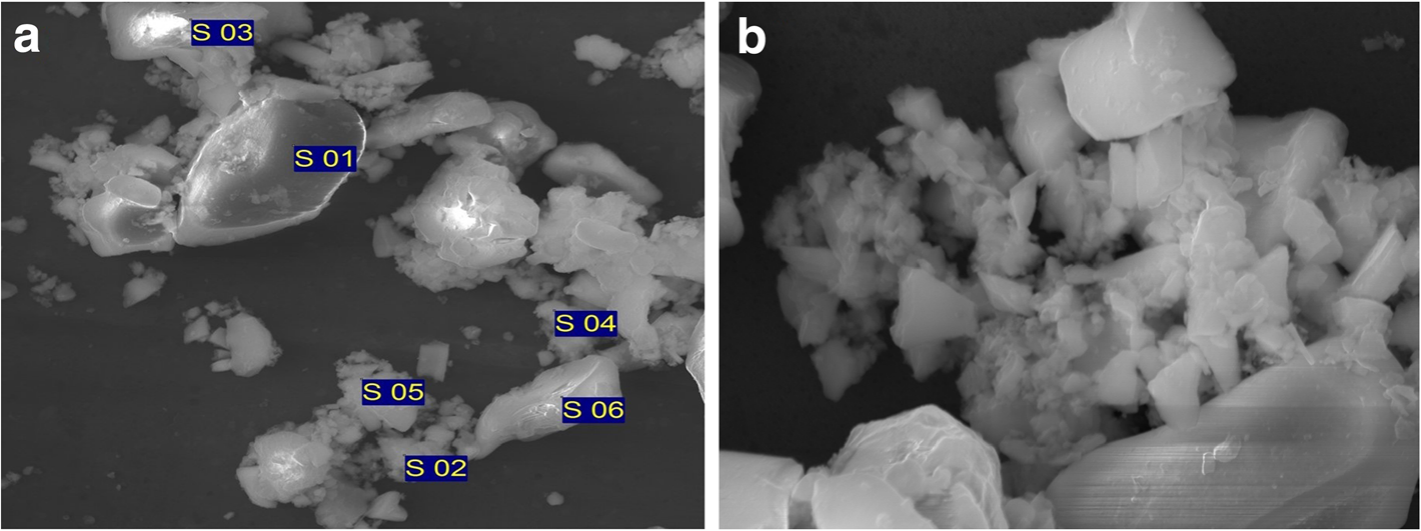
SEM-images of fine ZnS:Cu, obtained by SHS method. **a** General view. **b** Mixture particles of different sizes

Typical SEM images for fine fractions after rinsing in distilled water are presented in Fig. 1. As seen from SEM images synthesized fine fraction represents itself mixture of particles with different sizes, in which nanoparticles are present and as well particles of micro- and submicron sizes.

**Fig. 2:**
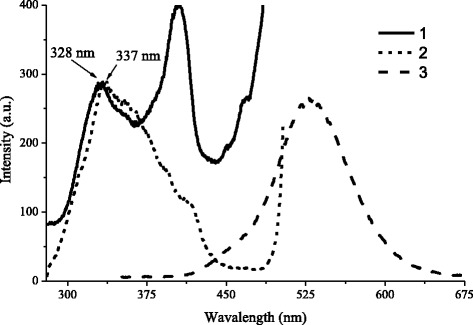
PLE spectra of SHS-synthesized ZnS:Cu, normalized on the intensity fundamental absorption band. 1 – initial ZnS:Cu – SHS, 2 – ZnS:Cu – SHS after washing in distilled water. 3 – PL spectrum of initial ZnS:Cu – SHS

In Fig. 2, PL and PLE spectra of ZnS–SHS are presented. As is seen from Fig. 2, PL spectrum of ZnS–SHS, represents itself wide band with maximum in 505–525 nm region. It is well known, that PL band of ZnS:Cu in blue-green region is complex and, as usual, represents itself the superposition of several bands, which are determined by copper admixture and also by intrinsic defects of ZnS. Nature of luminescence centers, which determine blue and green bands of Cu in ZnS, is described in detail in [6–8]. Authors of [6, 7, 9–13] have shown, that center, which is responsible for the appearance of Cu green band with λ_max_ ~ 505÷530 nm, is isolated copper ion, which substitute zinc ion in ZnS lattice.

Blue band with λ_max_~440÷465 nm connect with the formation of associates similar to DA pair type Cu_i_ -Cu_Zn_ [6, 7, 13] or Cu_Zn_ - Cu_Zn_ [11, 12]. In the given spectral region, the bands are also present which are caused by oxygen centers [6, 10, 14], and bands of self-activated radiation of [6, 7, 11, 12].

PLE of ZnS:Cu–SHS (Fig. 2, curve 1) includes bands corresponding to the band to band excitation transitions in bulk and quantum-sized ZnS. This indicates the presence in the material of large particles with sizes more than hundreds of nm and as well of the particles with sizes smaller than 5 nm (that is of exciton Bohr radius in ZnS). According to [14], E_g_ value for ZnS consists ~3.65 eV, which corresponds to the 340 nm absorption band. Shift of maximum of fundamental absorption band on 10 nm, tells about the presence in material of the particles with sizes corresponding to the manifestation of quantum-sized effect [15].

After washing of initial material in distilled water, the maximum of fundamental absorption shifted to the long-wave side (Fig. 2, curve 2). This testifies that the washing in distilled water has lead not only to the erosion from material of NaCl which remained after synthesis, but also and the particles with sizes smaller than 5 nm.

According to the data of scanning electron microscopy (see Fig. 1), the main part of the obtained material consists from two isolated fractions. First fraction consists from individual particles with sizes ~10 μm (Fig. 1a). Second part is represented by particles with sizes from hundreds nanometers up to several microns (Fig. 1b).

Thus, the chosen composition of charge and synthesis regimes enabled to simultaneously obtain, that is, during one synthesis, the particles with nano-, meso-and microsizes.

Investigations of element composition by EDS have shown, that in obtained ZnS:Cu in different particles the violation of stoichiometry can consist up to 4 at. % (Table 1).

**Table 1:** Element composition of ZnS:Cu particles, obtained by SHS method

Spectrum	Size, μm	Content, at. %		
		S	Cu	Zn
S01	12.7	49.07	–	50.93
S02	0.8	50.63	0.83	48.53
S03	8.9	53.51	0.21	46.28
S04	2.1	52.88	0.86	46.26
S05	0.4	48.53	0.90	50.57
S06	6.7	52.25	0.25	47.50

**Fig. 3:**
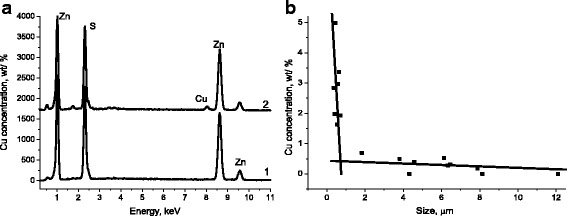
**a** EDS spectra of ZnS–SHS particles with sizes: 1–27 μm, 2–0.3 μm. **b** Concentration of Cu in dependence of ZnS–SHS particles sizes

At the same time, according to the data of these investigations, the presence of copper dopant in particles with sizes of order of hundreds nm is of order of ~2 wt.% (Figs. 3a, 2), and in large particles with sizes ~10 μm the presence of Cu was not detected (Figs. 3a, 1). In Table 1, the element composition (in at.%) of particles in conglomerates and of separate large particles shown in Fig. 1a is presented.

In Fig. 3b Cu concentration in dependence on ZnS-SHS particles sizes is shown. As seen from Fig. 3b for particles with sizes from ~300 up to ~700 nm anomaly high concentration of copper dopant is observed, with value ~5 wt.% for particles sizes 370 nm. At the same time for particles with sizes from ~2 up to 12 μm copper concentration consists not more than 0.7 wt.%, and in some particles (with sizes ~8 μm, ~12 μm) copper dopant is not registered by EDS method, that is, it is practically absent. It is necessary to note here that copper concentration in charge consisted ~1,5 wt.%. Thus, as testify mentioned above data, doping by copper of ZnS particles, which are formed during synthesis process is realized in different way depending on their sizes.

Let us consider the reasons which can explain such low level of doping characteristic for big particles (2÷12) and extremely high doping level for small (<2 μm) ZnS:Cu – SHS particles.

In our case, ZnS formation (materials with ion-covalent bonding, percentage of ionic bonding ~40% [15]) is realized with proximate neighborhood with NaCl (ionic material [16]. Appearance of ZnS phase during synthesis process undergoes with heat release, that in its case leads to the decomposition of CuCl on the components and NaCl melting. Melting temperature of NaCl - 800 °C, boiling temperature - 1465 °C [17]. Decomposition of CuCl on components undergoes due to disproportionation reaction, which leads to the formation of CuCl_2_ with release of Cu at 500 °C [18], and at temperature increase upper than 500 °C, CuCl_2_ in its case is decomposed on CuCl and Cl with subsequent volatilization of Cl. That is, crystallization of ZnS undergoes from melt of NaCl and ZnS mixture. Such type processes during transport in solid-liquid systems are called mineralization [1, 19].

Thus, the formation of ZnS particles with different sizes undergoes in parallel. Big ZnS particle are formed in melt or in NaCl surrounding. Copper transport through liquid phase of NaCl difficult due to its low solubility [20], besides that, CuCl decomposition undergoes not in one-time, and that also lows rate of Cu introduction into ZnS.

Formation of small-size particles, most probably, is realized in gas phase of Zn and S. In this case, appearance of CuS phase has low probability, because for its formation bigger quantity if heat is necessary [20]. After formation small particles are in neighborhood with Cu and big ZnS particles. Because the size of small particles becomes commensurate with the size of space-charge region (SCR) in ZnS, then the particles are in the region of charge carrier depletion. This is due to the fact that when the particle size decreases up to the value smaller than double value of screening depth (*L*) imposition occurs of SCR localized along one surface of particle on the SCR of opposite surface. Mutual overlapping of SCR surfaces leads to decrease of width between Fermi level *E*_*F*_ and top of valence band *E*_*V*_ [21]. Consequently, concentration of main charge carriers in particle with size *r <* 2 *L* turns out low, or in other words, particle turns out in depletion zone. In this case, as mentioned in [3], when impurity band is filled more than half first-order phase transition, accompanied by abrupt change of chemical potential value and other thermodynamic parameters of material, is thermodynamically favorable. In opposite case transition is accompanied by the increase of Fermi level during heating, which corresponds to the negative entropy of process, that is the decomposition of homogeneous material must be observed into the regions enriched with carriers to a concentration ensuring the filling of the impurity band not less than half and on regions depleted by carriers. In other words, the lowering of Fermi level and its entry into the zone of impurity levels leads to their emptying, that is thermodynamically unfavorable for material. Consequently, introduction of copper as donor impurity into ZnS, where copper occupy interstitial position becomes thermodynamically favorable for material. Possibly this has led to such high concentration of copper in the particles of small fraction. Also, for more unambiguous interpretation of the obtained results, the additional investigations are necessary.

Thus, the presented results show that SHS method enables to obtain materials with ZnS particles sizes in wide range - from micro- up to nanosizes. Besides that, the choice of synthesis regimes, flux material, and its quantity in mixture enables to selectively dope particles depending on their sizes.

## Conclusions

The carried out investigations of ZnS:Cu obtained by SHS method with adding of NaCl in charge as flux have shown, that introduction of NaCl enables to increase quantity of fine fraction (50–500 nm) in material. EDS data have shown, that concentration of Cu in fractions is different. Cu concentration in fraction with particles sizes within 50–500 nm consists ~2 wt.%, and in particles with sizes ~10 μm and more the Cu presence was not detected. Thus, selection of synthesis regimes, flux material and its quantity in charge enables when using SHS method to realize selective doping of particles in dependence of their size. The results obtained are well explained within the frames of the impurity «retrograde solubility» model in multicomponent materials.

## Abbreviations

*CuCl*: Copper chloride
*NaCl*: Sodium chloride
*SHS*: Self-propagating high-temperature synthesis
*ZnS*: *Cu* ZnS doped by Cu
*ZnS*: *Cu–SHS*-ZnS:Cu obtained by SHS method
*ZnS*: Zinc sulfide
